# Global movements need global leadership: expanding neurodiversity-inclusive education beyond Western-centric perspectives in Southeast Asia

**DOI:** 10.1080/20473869.2025.2527252

**Published:** 2025-09-12

**Authors:** Matthew Harrison, Jo Mosen

**Affiliations:** The University of Melbourne, Melbourne, VIC, Australia

**Keywords:** Neurodiversity, inclusive intervention, sustainable development goals, Southeast Asia, critical disability studies

## Abstract

This point of view article draws upon the lived experiences and professional collaborations of the authors to explore the opportunities and gaps in advancing neurodiversity-inclusive education across Southeast Asia. While the neurodiversity paradigm has gained momentum globally, much of the academic discourse remains centred on Western perspectives, often marginalising locally grounded approaches and innovations emerging from the region. Through our partnerships with researchers, advocates, and neurodivergent peers in countries such as Taiwan and the Philippines, we observe powerful examples of community-driven inclusion that remain largely invisible in the international literature. We argue that Southeast Asia offers rich, culturally grounded frameworks for understanding and enacting neurodiversity, shaped by collectivist values, relational care, and lived experience. However, these contributions are underrepresented in scholarly forums, limiting their impact on global policy and practice. By platforming regional voices and challenging Western-dominated narratives, we call for a more inclusive and pluralistic vision of neurodiversity that reflects the diversity of human experience across cultural contexts. This article highlights the urgent need to amplify Southeast Asian leadership in neurodiversity research and practice, and reflects on the ethical responsibilities of researchers to support, rather than overshadow, these emerging movements.

## Introduction

Neurodiversity is now understood as an umbrella term that recognises the broad range of ways human brains function, learn, process, and interact with the world (Chapman 2021). While much of the academic literature in this space continues to centre on autism and Attention-Deficit/Hyperactivity Disorder^1^ (ADHD), the neurodiversity paradigm as it is conceptualised today encompasses a far wider array of neurotypes. These include dyslexia, dyspraxia, dyscalculia, Tourette syndrome, intellectual disability, and acquired neurodivergence such as traumatic brain injury or neurological illness (Clouder et al. 2020). What binds these experiences together is not a shared set of traits or needs, but a shared history of marginalisation within systems designed around narrow ideas of what is ‘typical’ or ‘normal’ (Chapman and Bovell 2022). We join with many of our colleagues in arguing that this re-centring of knowledge around the lived experiences of neurodivergent people is not just a welcome development, but one that is long overdue (Botha and Gillespie-Lynch 2022; Fung et al. 2022; Kapp 2020). For too long, understandings of neurodivergence have been dominated by clinical authority. The neurodiversity movement insists that those most affected must be at the centre of conversations about inclusion, belonging, and justice.

These objectives of inclusion, belonging, and justice for neurodivergent learners are universal values. Centring neurodivergent voices in reconceptualising formal and informal systems of education is the essence of the United Nations Sustainable Development Goal 4, which calls for inclusive and equitable quality education and lifelong learning opportunities for all (UN DESA 2023). This goal cannot be realised without recognising and addressing the systemic barriers faced by neurodivergent students in educational settings around the world, including in Southeast Asia. Ensuring that learners of all neurotypes are understood, respected, and supported to thrive is not only a matter of equity, but a matter of fundamental human rights. When education systems overlook or marginalise neurodivergent individuals, they fall short of their obligations under this global agenda. Leadership is required to address this, drawing on both local knowledge and an understanding of the global body of research around inclusive education.

To be frank, we need a greater diversity of voices to sustain the neurodiversity movement, particularly the voices of leaders from Southeast Asia. As researchers in neurodiversity, we know that changing systems requires people with a range of responsibilities coming together and working towards a common vision. Teachers, school leaders, researchers, community advocates throughout Southeast Asia can and are already leading change at local, national and international levels. Most importantly, we argue that people in Southeast Asia with a direct lived experience of neurodivergence need to be allowed and empowered to serve in these leadership positions to truly bring the vision of the neurodiversity movement to life. Non-Western researchers such as De and Basu (2025) are starting to highlight regional examples of neurodiversity-affirming initiatives, but this research needs to be recognised by the global neurodiversity community. It is time that neurodiversity researchers in Western countries look to the expertise of leaders in Southeast Asian countries and ask ‘How can we make celebrating neurodiversity a truly global movement, and what can we achieve together? ’

## The rise of the neurodiversity paradigm and movement

At its heart, neurodiversity is not a clinical concept but rather a political and social movement. It seeks to reframe so-called deficits as neurological differences, advocating for respect, inclusion, and the dismantling of systemic barriers (Chapman and Bovell 2022). While it is easy to become complacent that this is a universally accepted position, we are still encountering educators and researchers who do not fully grasp the implications of this movement. This could be due to the origins of the term and the movement. Although the term has increasingly been taken up in academic discourse, often by neurodivergent scholars, it originated in grassroots activism. In the early 1990s, online bulletin boards became spaces for neurodivergent people to connect, exchange stories, and articulate a shared vision for change (Botha and Gillespie-Lynch 2022). While these early conversations were largely driven by autistic individuals, the call to shift the narrative from one of pathology to one of diversity resonated with people across a wide range of diagnostic categories (Botha et al. 2024; Chapman 2021). The neurodiversity movement builds upon the foundations of the social and human rights models of disability, both of which reject the idea that impairment alone explains exclusion (Lawson and Beckett 2021). Instead, they point to the role of social structures, attitudes, and environments in creating disadvantage. This has inspired a wave of lived experience-led advocacy across multiple domains, including education and education research, where neurodivergent people are increasingly stepping into leadership roles to reshape practice and policy.

We argue that a neurodiversity-informed approach, one that values cognitive differences as part of human diversity, offers a powerful transcultural lens through which to reimagine inclusive education in line with the ambitions of Goal 4. This approach moves beyond surface-level accommodations to interrogate how systems, practices, and policies can be transformed to support the full participation and flourishing of every learner, regardless of how they think, communicate, or engage with the world.

This also poses an opportunity to rethink the function of data ecosystems and a shift from reporting to system-wide transformation (UN DESA 2023). General Comment No. 4 to the UNCRPD requires signatories to create an enabling environment through a whole systems approach to inclusive education where implementation is the responsibility of the whole education system. A whole systems approach entails strong national policies and systems to support implementation of those policies.

Through our interactions with colleagues and friends across Southeast Asia, we are observing the quiet but powerful growth of neurodiversity-affirming communities and advocacy, often led by neurodivergent individuals and families themselves. However, these movements remain largely invisible in the academic record, in part due to the Western-centric gaze of most critical disability studies journals and the dominance of English-language scholarship (Cheng et al. 2023). This invisibility obscures the rich, localised ways in which neurodiversity is being reinterpreted and enacted in Southeast Asian contexts, often in ways that resonate with Indigenous, community-oriented values disrupted by colonial and medicalised frameworks.

## The challenges of hearing non-Western voices in the literature

Although the neurodiversity movement emerged as a call for equity and inclusion, much of its global discourse has been shaped by Western-centric perspectives. The literature that dominates educational research and policy is largely produced in North America, Europe and Australasia, reflecting the contexts, challenges and advocacy efforts of those regions (Cheng et al. 2023; Hirota, Cheon, and Lai 2024). While this body of work has undoubtedly advanced understanding and practice, it often overlooks or marginalises contributions from the global South. As a result, the neurodiversity movement can appear to be Western-led and Western-defined, limiting its perceived relevance and excluding alternative ways of understanding and enacting neurodiversity-inclusive education.

At the same time, we are seeing and hearing that the neurodiversity movement is gaining strong traction across Southeast Asia, where diverse communities of educators, advocates and neurodivergent individuals are leading transformative work. In fact, we argue that some Southeast Asian jurisdictions seem to have developed inclusive education frameworks that are more progressive and systemically embedded than those in parts of Australia, where we, the authors, are based. Yet, despite this innovation and leadership, efforts in the region remain underrepresented in the most cited academic journals, which continue to prioritise perspectives from Western institutions. It is perhaps understandable that researchers in these regions focus on their own local contexts, particularly given the significant challenges faced by neurodivergent individuals in their own systems. However, this inward focus has led to an incomplete global narrative that fails to recognise the full extent and diversity of the neurodiversity movement.

This imbalance has consequences. When the international discourse fails to reflect the work being done in Southeast Asia, it risks reinforcing the perception that the movement lacks relevance or legitimacy in the region. This perception can serve to justify inaction, not because of a lack of local will or capability, but because of the continued dominance of Western research in defining what constitutes ‘valid’ practice. We believe that it is essential that Southeast Asian researchers and practitioners are given space in international journals with high readerships to share their insights, approaches and critiques. This point of view article highlights two such examples, sharing brief vignettes of local initiatives that are contributing meaningfully to neurodiversity-inclusive education. We hope to expand the narrative and honour the leadership emerging from Southeast Asia.

## Matt reflecting on being not so uniquely human: a vignette of shared experiences with higher education colleagues in Taiwan

During my recent visit to National Taipei University (NTPU) in Taiwan, I had the privilege of sharing research from the University of Melbourne Neurodiversity Project, where I co-lead our efforts to support neurodivergent students and staff. As a visiting scholar, I was invited to present findings grounded in lived experience, highlighting both the barriers and opportunities for inclusion in higher education. After my presentation, an early career academic from NTPU’s Faculty of Sociology shared their own research into the experiences of neurodivergent students in Taiwan. Their work, situated within a broader movement for disability rights in the region, reflected a clear and growing commitment to neurodiversity-affirming education.

The first step in implementing neurodivergent-affirming education in any country is understanding the experiences of the local neurodivergent communities. As in my own country, there was still very little research at a university-level of the exact populations of neurodivergent people, including the numbers of neurodivergent students and their diagnostic labels, their experiences of studying at the institution and the types of accommodations or adjustments that would make their lives easier. In a fateful instance of academic happenstance, my research team and the researcher at NTPU had both tried to address this paucity of knowledge about the neurodivergent communities in our home institutions. When my research team and the researcher from Taiwan compared the datasets from our respective student needs analyses, we began to see that despite our shared perceptions that Australia and Taiwan had different cultural attitudes to neurodivergence, the ‘on the ground’ experiences of students were very similar.

While both of these studies are in the process of being published, these data exchanges reminded us that the aims of the United Nations Sustainable Development Goal 4, to ensure inclusive and equitable quality education and promote lifelong learning for all, are being advanced in culturally distinct yet deeply connected ways. There are many shared experiences, and from these experiences are opportunities for collaboration and collective learning. Despite our cultural differences, our findings echoed one another in striking ways. Neurodivergent students and staff in both universities spoke of social isolation, a deep longing for community, and a need for educational systems that allow them to be open about their strengths, interests, and challenges. They described the emotional weight of masking, the fear of disclosure, and the transformative potential of being met with understanding rather than judgement. What they were asking for was not extraordinary, just the right to be seen, heard, and supported on their own terms. For me, this reinforced the idea that inclusion cannot be reduced to policy statements or isolated adjustments. It must be embedded in the culture and structures of our institutions, creating spaces where neurodivergent people are not only accommodated, but welcomed and valued.

What stayed with me most from my time at NTPU were the conversations that followed. Speaking with researchers, staff, and students, I was struck by how personal this work is for so many of us. We are driven by stories—our own, and those of people we care deeply about. Together, we are working not only to open the doors of our universities, but to redesign them entirely, so that neurodivergent people can flourish as full members of our communities. Paraphrasing the eloquent words of Prizant and Fields-Meyer (2022), our movement is about honouring the uniquely human drive for connection and expression, on our own terms, in our own voices, and in spaces that embrace us as we exist.

## Jo reflecting on her time with J: a vignette of understanding the experiences of neurodivergent students in the Philippines

My professional and personal connections with the Philippines have enhanced my appreciation of cultural responses to diversity and inclusion. Most recently, my engagement with Our Lady of Fatima University has enabled a mutual exchange of knowledge informing neurodiversity practices. At a personal level, I have had the privilege of spending time with J, a delightful 10-year-old autistic girl who is part of my extended Filipino family. During family gatherings in both the Philippines and Australia, what strikes me every time is the seamless, intuitive way her family provides support. Her parents, grandparents, aunts and uncles move around her like a beautifully improvised circle of care; never orchestrated, but always attentive. Her father closely follows as she dashes ahead, while her mother carries a carefully curated bag of books, toys and craft materials selected by J to keep her engaged throughout the day. At any moment, one of her aunties or uncles gently steps in to spend time with her; painting, reading, or simply sitting beside her with one of her favourite toys. There is no need for formal instruction, no imposed structure. What unfolds is a living example of relational, responsive inclusion grounded in deep cultural values of connection, reciprocity and collective care. What is most evident is the role of collectivist cultures (Page et al. 2023), that provide a structure for neurodiversity to be valued and embraced.

As an educator and researcher, I find myself constantly reflecting on how J would be interpreted within formal education systems, particularly those that rely on medicalised or categorical assessments of disability. Her presentation of autism varies from moment to moment, sometimes indistinguishable from her peers, other times shaped by sensory needs, emotional responses or environmental shifts. She cannot be neatly placed on a spectrum ranked from mild to severe, yet these are the terms I often see applied to autistic children in school systems influenced by frameworks such as the Washington City Group questions (Groce and Mont 2017). These deficit-oriented models not only fail to capture the complexity of children like J, they risk obscuring the richness of the familial and cultural knowledge that supports her flourishing. They reduce a whole person to a set of functional difficulties, rather than asking how learning environments might adapt to meet her where she is.

Witnessing J’s experience has reinforced for me that the neurodiversity movement cannot, and should not, look the same in every context. In Southeast Asia, inclusion often grows from cultural values that prioritise communal responsibility, relational understanding and harmony within the group. These values offer profound lessons for global conversations on inclusive education. When we shape policy, we must begin with identity, context and lived experience and not standardised measures that rank difference. Aligning with Article 8 of the UN Convention on the Rights of the Child, disability scholars and advocates have long recognised that every child has the right to preserve their identity (Szmukler 2015). For J, that identity is not just autistic, but deeply connected to her family, her culture and her way of moving through the world. Any model of inclusive education that hopes to be genuinely transformative must recognise this, and must honour the forms of leadership and care that already exist within communities.

A key message from both vignettes is the need to listen in order to understand, whether that is at an institutional level as in the Taiwan needs analysis example or at a more intimate level as described in the story of J in the Philippines. To hear this stories, we need to create the conditions for leaders from these communities to be able to become valued voices in the international neurodiversity research and research-translation academy. This requires academics in Western countries to be active listeners, seeking out opportunities to work with and learn from these hidden voices, whether it be with an institution like NTPU or through co-design with people like J and their family.

## Reviewing UNESCO and other soft global policy perspectives

We also argue that we need to pressure our international bodies and globalling facing institutions to follow this trend to become better at listening to neurodiversity-affirming voices in Southeast Asia. A supportive and inclusive policy environment is essential for realising the aims of the neurodiversity movement across Southeast Asia, particularly in fulfilling the promise of Sustainable Development Goal 4: inclusive and equitable quality education and lifelong learning for all. Global institutions such as UNESCO play a pivotal role in this process by shaping the international agenda through non-binding but influential strategies known as soft policy. Soft policies are frameworks that influence behaviour through persuasion, cooperation and shared values, rather than through legal enforcement (Banerjee, Savani, and Shreedhar 2021). They rely on appeal and mutual interest to guide change. In the Southeast Asian context, soft policy has become a critical lever for advancing neurodiversity-affirming education, providing legitimacy and global visibility to local initiatives led by neurodivergent individuals, educators and advocates. The examples shared from Taiwan and the Philippines in this article illustrate how such policy environments create the space for culturally grounded, inclusive practices to emerge and flourish, guided by lived experience and collective care.

A prominent example of this influence is the work of the UNESCO Mahatma Gandhi Institute of Education for Peace and Sustainable Development (MGIEP), the first and only Category 1 institute of UNESCO located in the Asia-Pacific region. Fully funded by UNESCO and operating under its direct authority, MGIEP is a global leader in transformative learning research and education policy. Through its International Science and Evidence Based Education (ISEE) Assessment, MGIEP has generated critical insights into the design of inclusive education policies that take into account invisible disabilities and diverse learning differences, including dyslexia and autism. The institute’s work cautions against narrow, deficit-based interpretations of inclusion, and instead advocates for frameworks that honour the complexity of neurodivergent experiences. Such efforts reflect the growing awareness that inclusive education must be about more than access; it must enable meaningful participation, affirm identity and support learning across the lifespan. We see that these messages are beginning to shape national dialogues and regional collaborations in Southeast Asia, offering a powerful alternative to the dominance of Western clinical narratives.

In contrast, frameworks designed for national use in population-based surveys such as the Washington Group Questions (WGQ) (Washington Group on Disability Statistics 2020), risk marginalising rather than fostering inclusion. In recent times, Washington Group disability questions have been critiqued for their use of binary categories and severity rankings that reduce complex lived experiences to deficit labels. Bennani (2023) reports that these questions impose rigid boundaries and objectify disability, while Biermann and Pfahl (2020) note that they do not capture contextual barriers. Baart, Elbers, and Schippers (2023) report findings from their research of WGQ and broader disability self-identification categories, that WGQ measures often diverge from subjective self-identification in ways that unfairly inform policy and access.

In their attempt to quantify disability, the WGQ reduces the experience of disability into static, binary categories to define those with and without disability (Bennani 2023). Chapman and Carel (2022) articulate a neurodiversity perspective that honours human variation rather than reducing individuals to deviations from a normative scale of mild, moderate, or severe impairment. While aiming to make disability visible, the quantification process contributes to the production of new exclusionary rankings that limit the progress of the neurodiversity movement.

These statistical tools, often used to identify students for educational support, focus narrowly on functional limitations and observable behaviours, placing undue power in the hands of educators who may have limited understanding of neurodiversity. This approach risks flattening the complexity of neurodivergent identities and may silence voices like those of the Filipino child described earlier in this article, whose needs and strengths fluctuate in ways that defy categorical rankings. A policy environment that privileges global statistical uniformity over local knowledge and lived experience risks reinforcing the very exclusion that inclusive education seeks to dismantle. For these reasons, researchers from outside Southeast Asia have an ethical responsibility to not only collaborate with, but actively platform, local voices when writing about the region. Academics prioritising a diversity of geopolitical backgrounds at conferences and in publications, editors advocating special issues of internationally recognised academic journals with a focus on local voices within the Southeast Asia region, and leaders in Western institutions creating policies to support hiring non-Western scholars and advocates are all ways we can platform voices and to create the conditions for closer collaborations between Southeast Asia and other parts of the world. We contend that doing so is not simply an act of allyship. Rather, it is an essential step in building a truly global neurodiversity movement, led by those who live and shape it every day.

## Where next for the neurodiversity movement in Southeast Asia?

The immediate priority for advancing neurodiversity-inclusive education in Southeast Asia lies in platforming the voices and expertise of neurodivergent individuals and scholars within regional and international forums. Achieving Sustainable Development Goal 4, ensuring inclusive and equitable quality education and lifelong learning for all, requires us to centre those most affected by exclusion in shaping policies and practices. This is especially urgent given the invisibility of Southeast Asian contributions in global academic literature, despite clear evidence of grassroots innovation and culturally responsive practices throughout the region. Institutions such as the MGIEP offer critical pathways for amplifying neurodivergent voices through soft policy leadership. International journals and conferences must actively seek out and support scholarship from Southeast Asia, not as a gesture of inclusion, but as a recognition that these contributions are essential to a truly global neurodiversity movement.

In the medium term, regional collaboration and policy development must be prioritised to support sustainable systems change. National governments across Southeast Asia are already engaging with inclusive education in distinct and often progressive ways, with some jurisdictions demonstrating stronger commitments to system-wide equity than parts of Australia, where we are based. However, fragmented implementation and the continued influence of deficit-based assessment frameworks, such as those built on the Washington Group questions, risk undermining these efforts. Adopting a human rights-based approach to disability policy, grounded in the social model but attentive to individual contexts, can provide a shared foundation for reform. Strengthening regional partnerships between universities, ministries, and advocacy organisations will also be key to building capacity, sharing knowledge, and generating context-specific research that informs national strategies and aligns with the broader SDG 4 agenda.

Looking to the future, Southeast Asia is well positioned to lead globally in neurodiversity-affirming education across the lifespan, from early childhood through to higher education and employment. The region’s diverse cultural, linguistic and philosophical traditions offer unique opportunities to reframe dominant narratives of disability and difference. Collectivist values, such as those observed in Filipino and Taiwanese contexts, highlight strengths-based approaches to care, connection, and communal support that resonate deeply with the aims of the neurodiversity paradigm. As these local movements grow in visibility and confidence, they offer the potential to reshape global understandings of inclusion, not by replicating Western models, but by leading with practices grounded in relationality, respect, and belonging. Realising this future will require continued investment, strategic policy action, and above all, a commitment to centring the lived experiences and leadership of neurodivergent people throughout the region. We see Southeast Asia as being well placed to be a global leader and look forward to supporting our neurodivergent colleagues on this journey.

## Data Availability

Data sharing is not applicable to this article as no new data were created or analysed in this study.

## Note

1 Despite the diagnostic label referring to Attention-Deficit/Hyperactivity Disorder, we prefer the terminology Attention-Deficit/Hyperactivity Differences.
